# Digital Educational Escape Rooms for Providing Knowledge on Stress Management and Health Promotion for Students—A Rapid Review and Pilot Study

**DOI:** 10.3390/ijerph22010093

**Published:** 2025-01-11

**Authors:** Joanna Albrecht, Anna Lea Stark-Blomeier, Pascal Schütz, Nina Lenhard, Christoph Dockweiler, Pinar Tokgöz

**Affiliations:** Digital Public Health, Department Digital Health Sciences and Biomedicine, School of Life Sciences, University of Siegen, 57076 Siegen, Germany

**Keywords:** online escape room, digital game-based learning, health promotion

## Abstract

The impact of stress on students’ well-being and academic success is tremendous. This paper addresses the issue of balancing stress with the realm of a digital educational escape room (deER). This article demonstrates how a deER can serve as a means of providing knowledge on stress management and health promotion for university students. The objective was twofold—to explore the state of research regarding deERs in stress management and health promotion for students and to investigate the user experience and acceptance of a deER prototype. The methodology combines a rapid literature review and the conceptual as well as technical development of the deER prototype. Additionally, pilot testing was conducted in alignment with relevant theory. The rapid review included four publications meeting the inclusion criteria. Data for deER prototype testing were collected from students at the University of Siegen (first Bachelor’s and Master’s degree). The pilot study results (*n* = 4 participants) indicated that deER education on coping strategies, which incorporates mindfulness exercises, increases productivity and is considered helpful for stress management. This suggests that deERs can provide enjoyable and effective support for students in acquiring personal stress management strategies, potentially enhancing health promotion at universities. However, larger, more diverse studies are needed to fully assess their feasibility and integration into university structures.

## 1. Introduction

### 1.1. Impact of Stress on Students

Stress and psychological strain are widespread among students and can negatively impact their well-being and academic performance. According to health insurance data, in 2023, 44% of students in Germany will often experience stress during their studies or in private life, which is an increase of 23% compared to 2015 [1]. When compared internationally, this is well above average, with a recent meta-analysis showing a stress prevalence of 23% [2]. Examinations, additional work through part-time jobs, and pressure to perform well are identified as the main causes of stress [1]. Health interventions that promote resilience and address the stress management process of students should teach how psychological, social, and/or structural resources can be identified and (pro-)actively sought [3].

### 1.2. Educational Escape Rooms

Fostering an environment that encourages positive feelings in the educational setting is a tool that can improve academic performance, develop competencies, and enhance resilience in students [4]. However, there is still insufficient knowledge about how students with an increased risk of developing mental disorders due to stress can be effectively provided with knowledge regarding prevention and health promotion strategies. Educational escape rooms (eER) offer great potential to reach this target group. Educational escape rooms are defined as pedagogical activities that are time-constrained and problem-based and offer a good opportunity to address problem-oriented learning by developing tasks that are based on an authentic problem and embedded in a meaningful scenario [5]. Key features of an escape room activity are solving puzzles under time constraints in the context of a storytelling narrative. The word “escape” describes the spatial setting. The participants have to solve the puzzles to leave the escape room [6]. Methods used in educational escape rooms are collaborative or problem-based learning and gamification [7]. They can be distinguished from serious games in that the latter is also used for educational purposes but does not contain the concept of “escaping”. EERs have aroused the interest of the educational community since they can be applied in a wide range of academic contexts and provide an engaging learning environment for students [4].

### 1.3. Digitalization of Educational Escape Rooms

The use of digital technologies in the context of educational escape games is seen as effective and low-threshold—especially in post-COVID times, where digital games and applications have risen in popularity more than ever [8,9]. The development of digital teaching environments has introduced the concept of digital educational escape rooms (deERs) and gained attention as promising collaborative and playful learning experiences for higher education [10]. DeERs can be differentiated from digital games in that the latter includes many different types of games on digital platforms that can be used for pure entertainment and as educational tools but are not limited to the escape concept [11,12]. DeERs take place via web- or app-based applications in digital learning environments, making them more accessible. Research results show that this intervention can have significant positive outcomes on academic performance through improved motivation, positive behaviors, and engagement [13]. Current research shows that deERs not only promote cognitive learning but also support social and emotional learning outcomes while enhancing access to education. During the initial development phase of deERs, fundamental guidelines have already been drawn up, which will support their future evolution [7].

### 1.4. Study Aims and Research Questions

The lack of consistent evidence of deERs as a means to increase knowledge for the stress management of students warrants further research. To address this gap, our study explores the design, composition, and test of a deER for promoting stress resilience among first-year Bachelor’s and Master’s students in Germany. First, a rapid review of the current international research regarding the use of deERs in stress management and the promotion of health and well-being for university students will be presented. Second, we describe the development and results of testing a deER prototype. This objective was carried out by analyzing two research questions. First, how can deERs be used for the stress management and health promotion of university students? Second, what are the user’s experiences and acceptance of a deER prototype developed using the escapeED framework?

## 2. Materials and Methods

### 2.1. Rapid Review

Conducting a rapid review is useful when the completeness of the search is limited by time constraints, and available evidence needs to be summarized promptly based on current and relevant information. In addition, the rapid review focuses on the extraction of key variables and a simplified quality assessment, which ensures the relevance and practicability of the data obtained for rapid decision-making processes [14], which may be necessary in fast-moving research areas such as digital health interventions.

**Figure 1:**
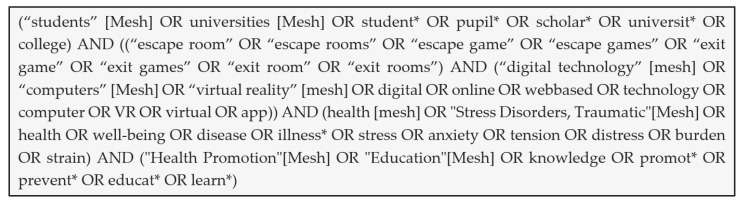
Search string for PubMed.

The rapid review followed the eight-step process outlined by Khangura et al. [15] to produce an evidence summary of digital educational escape rooms in stress management. *Step 1* is the needs assessment. The research topic was derived by the research associates from the needs and problems of the target group based on the literature. It was defined during the pilot study conducted with Master’s course students as the target group. In *Step 2,* the research question was developed and refined. An initial exploratory search was carried out. As it became apparent that there was a lack of literature on digital educational escape rooms in stress management for students, the research question was extended in terms of the promotion of health and well-being. *Step 3* includes the proposal development and approval. As the research project was not financially supported by a sponsor, no research proposal had to be prepared. In *Step 4,* the search strategy was developed, and a systematic literature search was conducted. We searched PubMed, Scopus, Psyndex, and ERIC. Table 1 shows the selected search terms according to the PICO approach (population, intervention, comparison, outcome) [16]. Boolean operators were used. The search string is shown below in Figure 1 exemplarily for the database PubMed.

A priori inclusion and exclusion criteria were defined, which also followed the PICO approach [16], summarized in Table 2. Due to the small size of the specific research field, no restrictions regarding the publication type or time were made. In line with the authors’ spoken languages, we restricted the search to articles in English or German.

*Step 5* of the rapid review process by Khangura et al. [15] is the screening and selection of relevant articles. The records from the database search were imported into Citavi to filter out duplicates. Due to time restraints, two authors (A.L.S.-B. and J.A.) shared the inclusion/exclusion process using the predefined inclusion and exclusion criteria and the software Rayyan. In the first round, the titles and abstracts were screened, and in the second round, the full texts. Uncertainties were discussed with a third reviewer (P.T.). A data extraction form was developed. One author (A.L.S.-B.) extracted general publication characteristics, e.g., author, year of publication, country, publication type/study design, and the primary study goal, on the one hand, as well as specific outcomes relevant to the description of the research field, on the other hand, e.g., the type of applied technology, the target group of the intervention, the health- or stress-related outcomes, the learning goals of the deER, the deER setting/story, and the use of models or frameworks in the escape game development. Two other authors (P.T. and J.A.) checked the completed table for correctness. *Step 6* followed the narrative synthesis of the included articles, comprising numerical summary analyses and qualitative content summaries. In the following *Step 7,* an evidence summary report was produced. The processing and publishing of the background, research questions, methods, and results take place within this article. This article and further publications will bring the new findings to the attention of stakeholders and decision-makers in science, policy, and practice within this field. This will enable, in the context of *Step 8,* an ongoing follow-up and dialogue with knowledge users.

### 2.2. Development of the deER Intervention

The strategic planning, implementation, and evaluation of health-related content was based on the public health action cycle (PHAC) [17,18]. The goal was to improve the stress resilience of students and provide knowledge about stress management strategies based on an evidence-based health promotion model. The combination with a game-based learning methodology, the escapeED framework [19], allowed for the integration of health-promoting content into a seriously playful approach to the deER. The process involves four phases: (1) assessing and recording the health problems of a population; (2) developing health policy intervention strategies; (3) implementing these strategies in the healthcare system; and (4) evaluating their acceptance and effectiveness [16]. The technical development of the deER followed a design-based research process and utilized the six steps of the escapED framework by Clarke et al. [19]. The precise implementation of the framework will be reported as part of the results.

### 2.3. Testing of the deER Intervention

**Figure 2:**
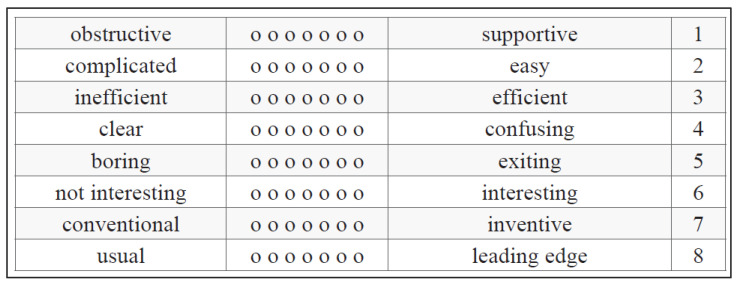
The short form of the UEQ-S [28].

The testing of the deER prototype was conducted on 1 February 2024 at the University of Siegen, Germany. An assessment was performed after completing the deER. For each participant, the following measures were collected:

- The subscale “stress level” of the stress and coping inventory (SCI) [20]: This assesses the stress level of the students (a 7-point scale from 1 “not stressed” to 7 “greatly stressed”; the mean value indicates the stress level of the students). Satow developed the scales of the SCI in German, which are reliable and valid for the measurement of stress-coping styles [21] (Satow L. 2012);
- A self-developed questionnaire for health consciousness based on Gould [22]: (A 5-point Likert scale from 1 “not at all reflecting my situation” to 5 “fully reflecting my situation”; the score indicates the level of health consciousness of the participants). A German version of this scale has been developed and tested regarding validity and reliability [23];
- The affinity for technology interaction (ATI) scale [24]: (A 6-point scale from 1 “does not agree at all” to 6 “completely agree”, with higher values of the sum score indicating a higher affinity for the use of technology). The 9-item ATI scale has been developed as an economic, unidimensional instrument for assessing ATI. Its validity has been confirmed, and its reliability is excellent [24,25];
- The system usability scale (SUS): This is a 10-item 5-point Likert scale (from 1 “strongly disagree” to 5 “strongly agree”); the total score is multiplied by 2.5 and indicates significant deficiencies (<50 points), good (>70 points) or ideal usability (=100 points), with a total score between 0 and 100 points, giving a global view of a subjective assessment of usability. To calculate the SUS score, first, the score contribution from each item has to be summed up. Each item’s score contribution will range from 0 to 4. For items 1, 3, 5, 7, and 9, the score contributions are the scale position minus 1. For items 2, 4, 6, 8, and 10, the contribution is minus 5 in the scale position. The sum of the score is then to be multiplied by 2.5 to obtain the overall value of usability. The score has a range of 0 to 100 [26]. The SUS is a widely used instrument that measures the subjective usability of products and systems, whereby its validity and reliability have been tested in various contexts [27];
- The short form of the user experience questionnaire (UEQ-S): Each item of the UEQ-S consists of a pair of terms with opposite meanings (see Figure 2).

Participants are supposed to rate each item on a 7-point Likert scale. The answers are scaled from −3 (fully agree with the negative term) to +3 (fully agree with the positive term). The first four items represent the pragmatic quality scale, and the last four items represent the hedonic quality scale. Values > 0.8 represent a positive evaluation, and values < −0.8 represent a negative evaluation [28]. For details concerning the design as well as validation of the UEQ-S, see [29,30]:

- A self-developed questionnaire for intention to use the deER based on the unified theory of acceptance and use of technology (UTAUT I) [31] (a 5-point scale from 1 “strongly disagree” to 5 “strongly agree”); the average score indicates the extent to which participants intend to use the deER in the future. The higher the score, the more likely the intention to use it. UTAUT I is an empirically tested validated model and is widely applied to examine the factors that might affect individuals’ adoption and use of technology in various settings [32];
- Sociodemographic data (e.g., age, gender).

First-year students from various Bachelor’s and Master’s degree programs at the University of Siegen were recruited for the pilot test. Initially, an attempt was made to recruit participants via notices posted in university locations such as the cafeteria or a digital bulletin board. However, due to a lack of response, active recruitment was carried out on the day of the pilot testing. Students were approached on-site, checked for eligibility, and informed about the pilot study. Those who were interested and gave consent were included as participants.

Upon obtaining consent from the participants, they were escorted to a seminar room, where they were provided with a notebook containing the deER. The study supervisor then provided instructions on how the piloting would proceed and how to operate the keyboard controls in the deER. Next to the notebook, a printed legend was placed listing the relevant keyboard controls for operating the deER. This served as a guide for the test person during the testing if required. The participants were encouraged to think aloud and verbalize their thoughts during the use of the deER (concurrent think-aloud) and after the use of the deER when answering brief follow-up questions from the study supervisor (retrospective think-aloud) [33]. The process was audio-recorded for subsequent evaluation. Additionally, another person participated as an observer throughout the entire testing. The recorded audio transcripts and observation protocols of each participant were used for qualitative analysis. Following the interaction with the deER, the participants completed a short questionnaire to assess their general affinity for technology and evaluate the usability and acceptance of the deER. The data collected from the short questionnaire were evaluated using content analysis.

The completion time of the questionnaires was approximately 20 min per participant. Descriptive statistics were employed to describe the sample characteristics and outcome measures.

## 3. Results

### 3.1. Rapid Review

**Figure 3:**
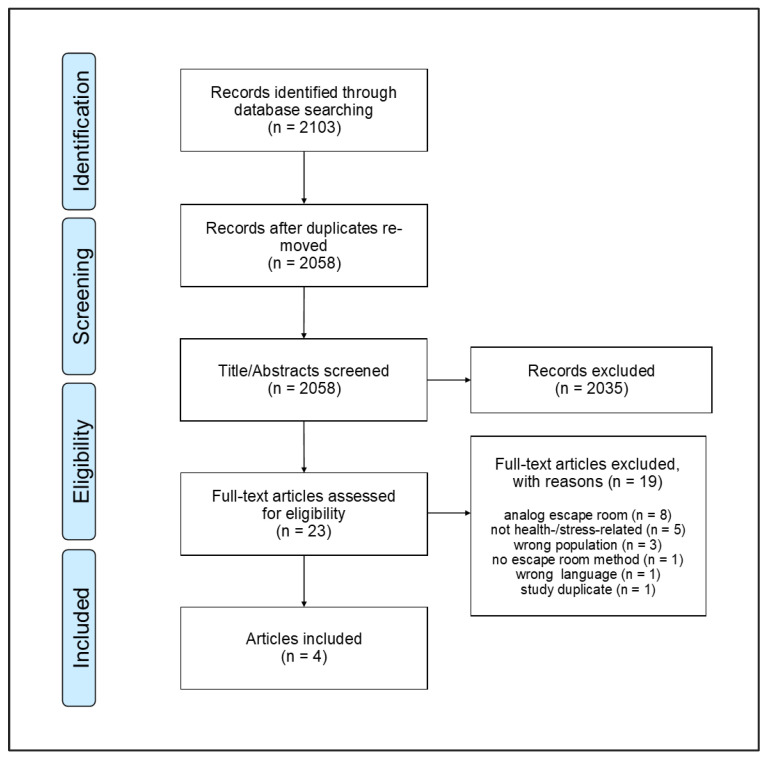
PRISMA flowchart.

The database search yielded 2103 articles, of which 45 were duplicates. Of the remaining 2058 articles, their titles/abstracts were screened. This resulted in 23 articles eligible for full-text screening. From these, 19 articles were excluded because the escape room was analog (*n* = 8), the study did not focus on health- or stress-related outcomes (*n* = 5), it was a wrong study population (*n* = 3), it had no escape room method (*n* = 1), it was in another language (*n* = 1), or it was a study duplicate (*n* = 1). Hence, a total of four articles were included. The PRISMA flowchart [34] is shown in Figure 3. The data extraction chart is provided in Table 3.

The paper of Labrosse et al. [35] from France is a cross-sectional study from 2024. The goal of this study is to describe the co-creation process of a digital escape room targeting healthcare students currently doing their internship at the health research center. All health-related fields of study (e.g., medicine, midwifery, and speech therapy) at the University of Bordeaux in France were included, recruiting a total of 45 participants. During the development process, the existing escape room, “Manage your emotions”, was adapted and renamed to “EscapeCovid”. The escape room was a multiplayer game for 4–6 players with digital synchronous interaction. It was developed by the startup Tricky to discuss the presented puzzle using computer cameras and headphones. The players are positioned in Thomas’ room, a fictional university student living in a shared flat and experiencing difficulties during the first COVID lockdown. Moving through the three rooms is possible by solving the puzzles hidden in different objects. It is designed in a linear structure; therefore, players can only move to another room if they solve all the enigmas. Players are led through the escape room by a game guide (a real person with expertise in the game) who explains the rules and answers questions. At the end, the guide concludes the game with a debriefing session. The co-creation process of the escape room follows the PRODUCES framework (problem, objective, design, end users, co-creators, evaluation, scalability) of Leask et al. [39], and it was designed with the help of the participatory co-design approach (using “collective creativity”) by Jessen et al. [40]. The escape room aimed to educate players about mental health issues, reduce the associated stigma, and equip them with strategies to address these challenges effectively. The sample of 45 students answered a questionnaire concerning satisfaction with the game and assessed the impact that the game had on mental health literacy. Additionally, a sample of ten students was interviewed about the gaming experience and its impact on mental health literacy. With 60%, most participants indicated that the deER made them understand the importance of talking about mental health, and 84% thought that the game increased their knowledge about mental health. The interviews revealed that the game enabled players to better understand and identify different emotions, but the interviews revealed that while the game helped the players better understand and recognize different emotions, it lacked content addressing the broader, holistic concept of mental health.

Moffett et al. [36] conducted a study in Ireland using a pre-test-post-test design to assess the effectiveness of the deER, “The Hidden Hospital”, designed for second-year medical students at the RCSI University of Medicine and Health Sciences in Ireland. The study’s primary goal was to explore how the deER could help students manage uncertainty during their transition to clinical placements. The target group consisted of 22 students enrolled in their second year of medical studies. The deER was a multiplayer online game for 4–5 players in a digital synchronous interaction. It was hosted on Microsoft Teams using Genial.ly platform, where the participants solved ten puzzles to “escape” a fictional creepy hospital setting. The game incorporated ambiguity and uncertainty, reflecting challenges in healthcare settings, such as managing complex information and navigating unpredictable outcomes. Support for the participants included pre- and post-game debriefs and technical assistance. The learning goals focused on helping students develop strategies for handling uncertainty, with particular attention given to teamwork and critical thinking. The health-related outcomes included enhanced uncertainty management and improved problem-solving abilities. While most participants felt they had learned valuable skills from the deER, some noted that the game did not fully reflect real-world clinical uncertainty. The quantitative results showed that 77% of the participants found the deER to be highly beneficial for learning, with 82% agreeing that it helped with uncertainty management. The study applied a design-based research (DBR) approach [41] and utilized the community of inquiry framework [42], emphasizing the social and cognitive dimensions of learning in virtual environments.

In the study by Sánchez-Ruiz et al. [37], the authors analyzed students’ opinions, feelings, and performances during and after playing the escape game using a cross-sectional design. A total of 296 students in the field of Aerospace Engineering (subject Mathematics I) at the Technical University of Valencia in Spain were recruited to use the multiplayer (3–5 players) game with face-to-face interaction. The online game was used via a digital platform in the same room using the Genial.ly platform and RPG Maker MZ software. The escape room consisted of individual games with no overarching story. The participants were able to choose their avatars. During the game, they could score or lose points based on performance by doing linear structured puzzles. The primary aim was to explore student emotions and learning outcomes. Students’ feelings were assessed using the RULER strategy following Nathanson et al. [43], which focuses on recognizing, understanding, labeling, expressing, and regulating emotions. Data on student opinions and feelings were collected through a questionnaire. The results showed that the predominant emotions were motivation (18.9%) and happiness (11.1%), while stress (7.4%) was also common but not viewed negatively by participants. The deER’s impact on learning was assessed through surveys, with most students indicating that the game reinforced their theoretical knowledge (76%), increased motivation (56%), and helped generate a positive emotional environment (55%). Additionally, 63% of participants felt that the deER contributed to the development and reinforcement of their knowledge. This study highlights the effectiveness of deERs in enhancing motivation and emotional engagement while also reinforcing academic content.

The fourth paper by Yllana-Prieto et al. [38] analyzes how the escape room affects multidimensional domains like attitudes, self-efficacy, and emotions of students. Students, or, respectively, pre-service teachers enrolled in a science course called “Knowledge of the natural environment in primary education”, studying in the senior course of the Primary Education degree at the University of Extremadura in Spain, were recruited (*n* = 42). The types of technology used were computers, tablets, or mobile phones that could be operated from the students’ homes. The game instructions were available on the university’s online platform Moodle, and communication took place via email and Zoom. The content focused on the solar system, renewable energy, and concepts such as the sun’s role in sustainable development. In the game, participants followed a narrative involving a letter from the scientist Carl Sagan, solving digital puzzles and locks to progress. A trainer provided support. The learning goals included understanding the solar system, planets, asteroids, and other related concepts. To investigate stress- and health-related outcomes, the study also assessed students’ emotional responses and stress levels before and after playing the game. The classification of positive and negative emotion groups followed Bisquerra [44]. Emotions like joy, satisfaction, and fun increased significantly, while confidence decreased. In contrast, negative emotions such as nervousness, frustration, and concern also increased. Significant changes were observed in six of the ten emotions analyzed. Correlations were found between positive emotions, attitudes, and self-efficacy, indicating that the deER positively influenced multidimensional learning outcomes.

### 3.2. Development of the deER Intervention

In the following, the development of the digital educational escape room as part of a Master’s course at the University of Siegen, Germany, along with the escapED six-step framework by Clarke et al. [19] with reference to the PHAC [17,18], are described.

#### 3.2.1. Step 1: Participants

The initial step was to analyze the target group and gather information about their needs and requirements for a deER [19]. An exploratory literature review and qualitative semi-structured interviews with students were conducted to gain a deeper understanding of the target group’s perception of stress. We also aimed to identify the target group’s knowledge requirements and needs regarding successful and unsuccessful coping strategies, as well as measures to reduce stress. Additionally, exploratory interviews served to explore previous experiences with deERs and to find out what (knowledge) needs the target group had regarding stress resilience during their studies. The target group for the deER comprised students at the University of Siegen who were in their first Bachelor’s or Master’s semester and were, therefore, encountering new structures. To ensure adequate segmentation of the defined group, the age range was set between 18 to 27 years, with the goal of achieving a balanced gender ratio. To achieve the objective of the target group analysis, three qualitative semi-structured interviews were conducted. The interviewees were selected from the student population of the respective working groups. The interviews took place at times and places chosen by the interviewee after an informative discussion and signing of the consent form. The interviews were audio-recorded, transcribed according to Dresing and Pehl [45], anonymized, and analyzed in preparation for the derivation of learning and game objectives of the deER. The qualitative data evaluation was conducted using content analysis and MAXQDA 2022 software.

#### 3.2.2. Step 2: Objectives

The relevant findings of the target group analysis were discussed in the Master’s course, and knowledge requirements, followed by the corresponding learning and gaming objectives, were identified (see Table 4). The interview material was used to identify the core knowledge needs of the students on the topic of stress management and stress resilience. A separate level in the deER was to be designed and developed for each core requirement. The analysis of the interview material revealed the two core knowledge requirements, “time management during studies” (level 1) and “strategies and internal university offers for coping with stress” (level 2).

Specific learning objects were derived for the two core requirements to operationalize the educational effect of the level. Gaming objects and implementation in the game were then defined for integration in the game. For this purpose, a variety of activities were developed that players can experience to achieve the defined learning objectives. To effectively engage the players, it was important to integrate narrative elements and specific content in a targeted manner. This is particularly important for deERs, which require a strong thematic and narrative depth despite the limited interaction time [19].

#### 3.2.3. Step 3: Theme

In this step, players’ motivations, game stories, and content were considered to create an engaging game experience for the intended players. The escapeED framework proposes to develop the theme of an escape room [19]. In this context, we chose a narrative design for the game to keep the players interested. To make the theme more engaging and realistic, the prototype was based on a fictional story that reflects a student’s everyday life during exam time when they are faced with more stress. As both levels took place in different virtual spaces (level 1 in the student’s premises and level 2 in the library), the two levels were linked by a storyline. Additional tasks were developed for this purpose (e.g., the search for a library book that must be returned). Also, the tasks in the individual levels were linked by further tasks (e.g., searching for a borrowed book from the library). The game ends when the student playing is asked to leave the library to meet up with friends after successfully learning. Finally, a script with the most important information from the entire deER can be downloaded from the laptop in the library.

#### 3.2.4. Step 4: Puzzles

**Figure 4:**
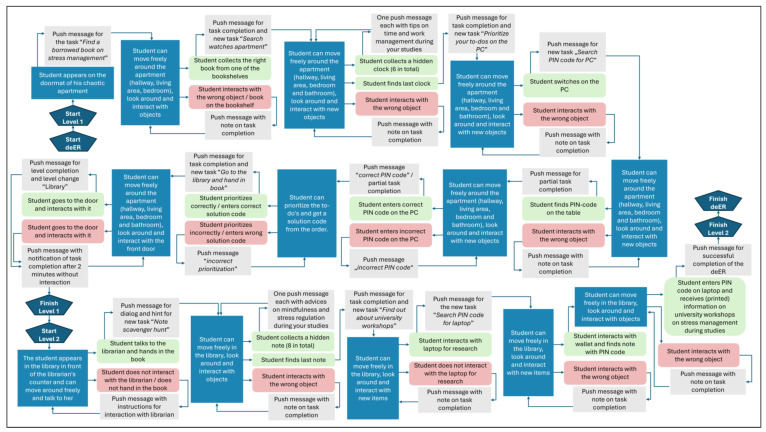
Simplified representation of the linear game logic of the deER.

The puzzles’ design required precise planning to achieve a clear objective, establish the context of the main storyline, and work out the spatial design and game mechanics in detail. Clear instructions, hints, coordinated visual designs, feedback, and rewards should guide the participants through the game to ensure a balanced gaming experience [19]. The structure of the linear game logic was planned based on the conceptual map example used by Greter et al. [46]. The linear logic of the deER is shown in Figure 4.

The deER was set up into two levels with linear flow according to the escape box theory. Players had to complete each stage’s puzzles and collect all the codes before moving on to the next and so forth until the final lock. In this study, each level was associated with a particular block of contents.

**Figure 5:**
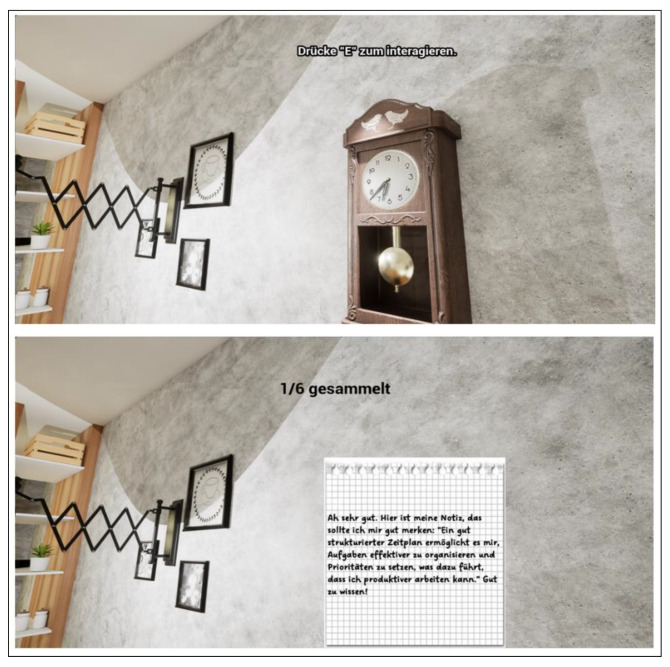
Excerpt level 1—Collect clocks and obtain information.

**Figure 6:**
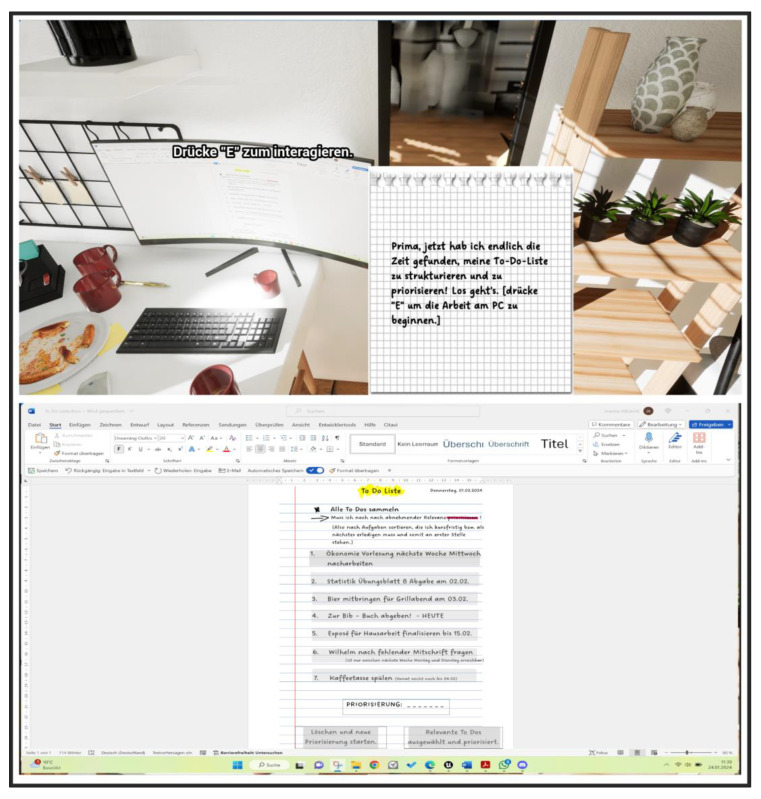
Excerpt level 1—Prioritize unimportant and important tasks.

**Figure 7:**
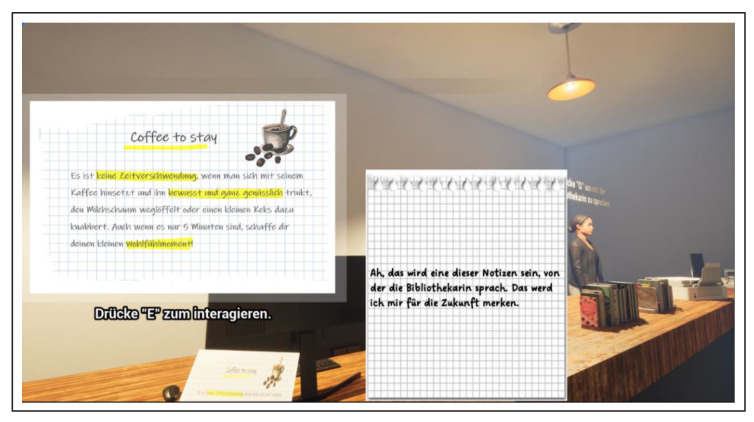
Excerpt level 2—Collect index cards with information on coping with stress.

**Figure 8:**
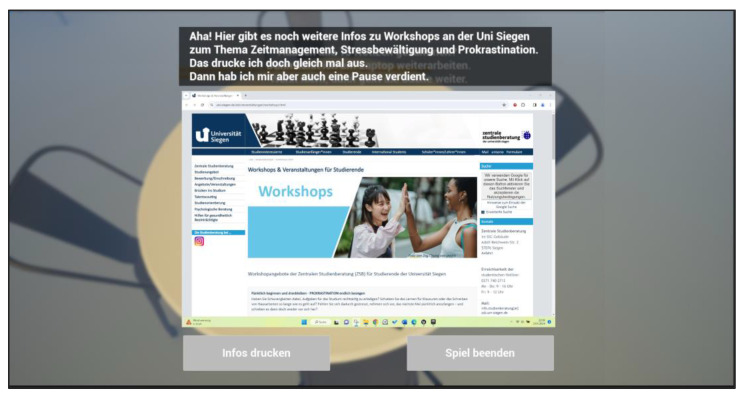
Excerpt level 2—Obtain information on internal university offers.

First, the players should decipher various aspects of time management to experience playful progress in the deER. Overall, the integration of rewards contributed to successful time management, whereby wasting time was declared a challenge. The design of the deER was based on a realistic learning environment for the players. Accordingly, the first level was designed in the style of a student-rented apartment, which is filled with learning materials and everyday objects to represent the student’s place of learning. The second level was designed in the style of a university library. A librarian has been integrated there, allowing interaction and introduction to the level and the puzzles it contains. The integrated puzzles are number puzzles, jigsaw puzzles, as well as search and solution tasks. The solutions to some tasks provide a didactic learning component that reflects how to deal with stress using mindfulness exercises that reflect the behavior of the players and create space for reflection. Furthermore, the interaction with objects such as a laptop provides a digital component, which shows the offer of the institution in the context of stress management to solve the deER. Concurrently, this offer is made available to the players in analog form to create added information value for future interests. The following figures provide an insight into the German-language version of level 1 (see Figure 5 and Figure 6) and level 2 (see Figure 7 and Figure 8).

#### 3.2.5. Step 5: Equipment

The deER was developed using the “Unreal Engine 5.3” software from Epic Games, a US-based company, and is digitally accessible through a notebook with a graphic card. The location of the prototype was to be a mobile experience.

#### 3.2.6. Step 6: Evaluation

This step was used to assess the overall usability and user experience of the study participants’ interactions with the deER prototype. The prototype was evaluated as a pilot test to ascertain its fundamental suitability as a means of promoting knowledge transfer on stress resilience, as well as to identify any further necessary developments of the gaming experience.

The participants in the pilot test were predominantly female, 25.5 years old on average, tended to be more technical affine, had a more pronounced health consciousness, and had a higher level of stress (see Table 5).

### 3.3. Testing of the deER Intervention

Four persons participated in the testing of the deER. Since this paper is a pilot study and has a limited sample size (*n* = 4), the significance of the results could not be calculated. Therefore, only the means and standard deviations were calculated and presented.

Related to the usability of the deER, the system usability scale shows moderate results (see Table 6). Regarding particular items, the participants would like to use the system frequently (Q1), thought the system was easy to use (Q3), and felt very confident using the system (Q9) during the study. The mean total score was 75.60 (SD 11.6), indicating that the overall interaction with the system was positive.

**Figure 9:**
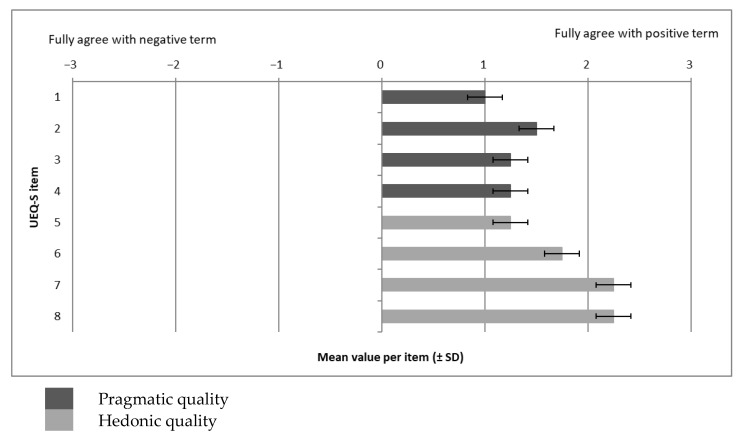
Results of the UEQ-S (*n* = 4).

The assessment of user experience revealed important aspects of the deER for providing information for the stress management of students during the study period. As seen in Figure 9, the results show an overall positive evaluation of the deER. The participants found the system to be inventive (M 2.30 ± SD 0.50) and novel (M 2.30 ± SD 1.00). They also assessed the deER to be interesting (M 1.80 ± SD 1.50) and easy to use (M 1.50 ± SD 1.30). On average, the results for the user experience show a consistently positive tendency, with the intervention being seen as very interesting, original, and novel compared to the other stress management strategies (e.g., reading, podcasts, or lectures).

**Figure 10:**
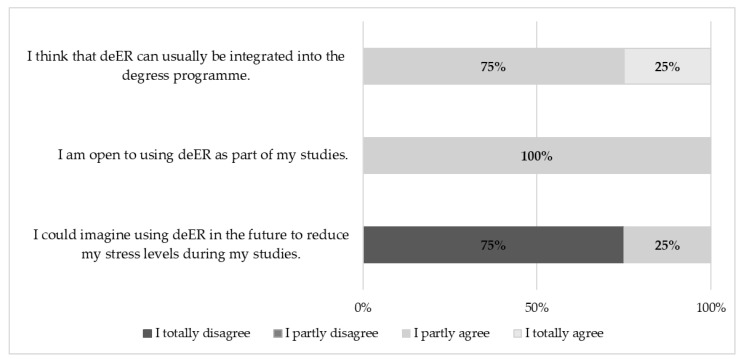
Results regarding intention to use a deER (*n* = 4).

In the context of intention to use the deER, the participants stated that they could not imagine using a deER in the future to reduce their stress levels during their studies (75% totally disagree). However, the respondents indicated that they are open to using deERs as part of their studies (100% partly agree) and that deERs can usually be integrated in the future to reduce stress levels during their studies (75% partly agree, see Figure 10).

The four observation protocols of the respective testing showed that there were uncertainties regarding the individual tasks, and the interaction with the objects was not always intuitive, especially for the participants without experience using a deER. In addition, difficulties arose in particular when identifying the clocks in the first level and collecting the individual notes in the library in the second level. When evaluating the open questions regarding the particular elements of the deER, the respondents revealed different perspectives on helpful information or coping strategies that could be considered helpful in the future. One respondent mentioned prioritizing the to-do list, one respondent the use of breaks to increase productivity, one respondent the body scan, and one respondent the general mix of information and play.

## 4. Discussion

### 4.1. Rapid Review

This rapid review aimed to investigate the current international state of research regarding the use of a deER in stress management and health promotion for university students. We found four publications, one of which deals with the promotion of mental health in the context of the COVID pandemic, and another refers to the management of uncertainty during the transition from class to clinic for healthcare students. The other two articles deal with teaching content in non-medical study programs while examining the influence of the deER on students’ emotions (e.g., stress or nervousness). It can be seen that deERs for promoting health and well-being are already being used in health-related and non-health-related degree programs.

This rapid review suggests that deERs are still rarely used to disseminate health-promoting knowledge, and those that are used in this context are based on different procedures or frameworks. Two of the reviewed studies did not report using a health-, game-, or learning-related theory, model, or framework regarding the intervention’s development.

Since the goal of this type of intervention is to reduce stress and negative emotions, this could be counteracted using targeted strategies to support students. Based on the review results, for the further development of digital interventions regarding stress management and health promotion among students, especially through digital escape rooms (deERs), it should be considered that such games can also evoke negative emotions such as nervousness and frustration [38]. To counteract this, targeted strategies to support emotion regulation could be integrated directly into the game, for example, through specific feedback mechanisms.

At the same time, other results show that stress in a deER is not always perceived as negative but can also have a motivating effect [37]. This suggests that stress can be specifically incorporated into the game design as a moderately stressful but motivating factor, whereby it should be ensured that the stress level remains controlled.

Regarding long-term teaching of learning content, the findings of Labrosse et al. [35] also indicate that content should be designed in such a way that a holistic understanding of the subject matter is best conveyed so that underlying concepts can be understood holistically and put into practice.

Following on from this, it can be concluded from the results of Moffett et al. [36] that scenarios that are as realistic as possible in the context of the learning object are necessary for the practical application of the learning content taught [36]. Thus, the realism of the implementation of learning content can be a central aspect.

However, it should be noted that the findings of this rapid review should be interpreted with caution due to the limitations of the reference population. It is well-established that the strategic use of theory-based methods can enhance the effectiveness of health interventions [47]. An examination of diverse frameworks is a viable avenue for further investigation, but there is still no framework that describes the development of deERs as health-promoting interventions.

Team-based and linear-conceptual deERs are frequently employed to enhance awareness of health-related topics and content in a motivating manner. However, it also becomes evident that the communication of content in a deER necessitates the formulation of a meticulous fundamental concept to facilitate the conveyance of knowledge in a manner that is accessible and comprehensible [35,36,37]. The complexity of the puzzles may also impede the transfer of knowledge. A team-based approach may prove beneficial in this regard, as it can help to mitigate uncertainty and frustration through the exchange of ideas and collective decision-making [36,38].

The following limitations should be mentioned when interpreting the results of the rapid review. Due to the limited time available, the screening of the articles was divided between two people. In case of discrepancies in the evaluation, an independent third party was involved to support consensus and ensure the objectivity of the results. Although rules for inclusion and exclusion were defined in advance, it cannot be guaranteed that these were not interpreted or applied differently by the two reviewers. In addition, the overarching term “health promotion” was used in the search. It is possible that not all relevant articles were identified, as more specific terms such as education, health behavior, risk factors, health literacy, or others were not used.

### 4.2. Development of the deER Intervention

The development of the deER intervention focused on the design and framework that would allow students to engage with stress management techniques interactively. To guide the process of development of deERs for health promotion, the escapeED framework was chosen. This framework integrates key elements of game-based learning, pedagogical theory, and the development process into a cohesive structure. Moreover, the use of the escapeED framework enabled the mapping of learning objectives against puzzles and narratives to build a cohesive interactive story that provides contextually immersive learning experiences. The framework intends to provide educationalists with a guideline to develop a deER. As there was no known framework that addressed the development of a deER as a health-promoting intervention, the choice of framework focused primarily on the reference to educational serious gaming mediation. Informed by Arnab and Clarke’s *Trans-Disciplinary Methodology for Serious Games Design* [48] and Nicholson’s white paper on entertainment escape rooms [6], the escapeED framework should be considered as a basis on which key elements of game-based learning, pedagogic theory, and development process are brought together.

Further work should address the validation and evaluation of the framework and whether it can be considered a useful tool for aiding the development of deERs for higher education on health-promoting topics with greater sample sizes and different fields of study, as well as in comparison with traditional learning methods.

### 4.3. Testing of the deER Intervention

This pilot study aimed to investigate users’ experiences and acceptance of a deER prototype as an innovative tool for providing knowledge for stress management and enhancing stress resilience of students in their first Bachelor’s or Master’s semester and thus in critical transitional phases of their studies. The results revealed that, after knowledge transference, coping strategies such as prioritizing to-do lists, incorporating mindfulness exercises such as the body scan, and taking short breaks to increase productivity are considered helpful in the future.

These observations are consistent with the results from the reviewed literature, which show a strong interest in offers to promote mental health, especially in resilience and mindfulness [1]. Interest in time and self-management, as well as physical health through exercise, is also present in the literature [1]. The findings from the literature stand in contrast to the results of the generalizability of the study conducted. These indicate that there are differences in stress and stress management depending on gender, type of university, type of degree, field of study, and federal state [1,49], which may not have been detected in this study due to a lack of variability between the participants. There are also marginal differences in the identified stressors compared to the literature. In this study, intrapersonal aspects (mainly own expectations and demands; life goals) were cited as stressors, followed by interpersonal or university-related stressors (pressure to perform). The latter are named as the main stressors in the literature [49]. Regardless of the results, the resilience of students can be positively influenced by various measures, with universities and students bearing equal responsibility for improvement [3]. The deERs offer opportunities in this context, particularly in motivation to change behavior [50,51].

The results of this pilot study should be interpreted with caution due to its limitations. The small sample size of only four participants from the target group who participated in the subsequent evaluation is a major limitation regarding the generalizability of the results. The small number of participants also increases the likelihood of bias and reduces the statistical robustness of the results. In addition, the small sample size makes it difficult to identify significant patterns or differences that may be relevant to a larger target group. The data collected in this study are, therefore, limited to a specific group of individuals. Furthermore, the recruitment efforts only resulted in the participation of students from the first Master’s semester of the Digital Public Health course at the University of Siegen. It was not possible to recruit students from other Master’s courses at other locations or from Bachelor’s courses. It should be noted here that the participation of students from various degree courses and programs could have led to different results. The cognitive interviews were limited to assessing usability and determining whether the use of a deER is a viable method for providing knowledge on stress management. The actual impact on health knowledge transfer could not be investigated in the pilot study and would require further research to make comparisons with control groups. It is important to consider the social desirability effects, as participants may not feel comfortable expressing their true views even when assured of anonymity [52]. Therefore, further data are necessary to validate these preliminary results. In addition, a potential side effect of general escape rooms that has been little investigated in the literature and practice is the possibility that participation in a deER could increase, rather than reduce, the stress levels of participants during completion. Future research should look more closely at this possible effect to ensure that a deER is targeted at stress-reducing elements and that potentially stress-inducing factors are minimized.

Based on the results of this comprehensive study, various recommendations for action can be derived for research, policy, and practice. This rapid review indicates that deERs have significant potential, although there has been limited research on their use in health promotion in universities. The use of frameworks and approaches from co-creation and learning theories in the development of escape rooms was interesting. In the future, it should be investigated to what extent their use influences the effectiveness of the games.

This pilot study indicates that deERs can offer enjoyable and beneficial assistance in familiarizing oneself with and acquiring strategies for personal stress management while studying. This could potentially create new opportunities for health promotion at universities. Nevertheless, a key result was that the participants wanted to adopt and apply individual pieces of information from the deER for their everyday lives, but they could not currently imagine using a deER to reduce their stress levels prospectively. Here, it would be beneficial to investigate which aspects of the deER facilitate the use of it for stress management during their studies and how it might be integrated into their learning environment. In this context, it would be appropriate to have various iterations during deER development to adjust it to the preferences of participants.

The results offer valuable insights into the feasibility and usability of a deER, as well as the acceptance of students in their first Bachelor’s or Master’s semester. However, to usually integrate a deER for students into university structures as a health-promoting offer, further research is required with larger and more diverse samples and more comprehensive study designs. The recommendations for future research are conducting further studies that have a larger sample size, testing different deER methods, taking place over a longer period of time, and including a control group that can identify possible causalities or measures of the learning outcomes. Future studies should be as interdisciplinary as possible, identify potential in this area, and define the various needs more precisely [1,49,51] while considering the constantly changing environment.

## 5. Conclusions

Promoting resilience is a long-term process that often depends on individual factors. To unfold the (supposed) potential of the digital approaches under consideration, a holistic, interdisciplinary approach of different specialist disciplines, for example, psychology, public health, and computer science, should be used. Overall, the deER developed is an example of DGBL. Despite some methodological shortcomings, such as a small and limited sample and insufficient internal consistency of some scales, the results encourage the use of deERs to impart health-promoting knowledge, on the one hand, and the conceptualization of a framework to develop deERs as a health-promoting intervention, on the other. This study demonstrates that deERs can be a tool for promoting health knowledge in stress resilience if designed appropriately. This approach can motivate students to engage in game-based learning on health-related topics as a part of their daily lives and as an integral component of university structures.

## Figures and Tables

**Table 1:** Search terms of the rapid review.

Item	Term	Synonyms
Population	Student	Pupil, scholar, university, college
Intervention (Part A)	Escape room	Escape game, exit game, exit room
Intervention (Part B)	Digital	Online, web-based, technology, computer, virtual, app
Comparison	Not Applicable	Not Applicable
Outcome (Part A)	Provide knowledge	Promotion, prevention, education, learning
Outcome (Part B)	Health	Well-being, disease, illness
Outcome (Part C)	Stress	Anxiety, tension, distress, burden, strain

**Table 2:** Inclusion and exclusion criteria.

Criteria	Inclusion Criteria	Exclusion Criteria
Population	Students in university or college	Other groups of people associated with university (e.g., teachers), students from other educational institutions (e.g., primary/secondary schools)
Intervention	Digital escape rooms for stress management or health promotion using different technologies (e.g., computer program, VR applications)	Other digital games or serious games that do not follow the logic or methods of an educational escape room or analog escape rooms
Comparison	Not Applicable	Not Applicable
Outcome	Health- or stress-related outcomes (e.g., knowledge about well-being, mental health)	Other outcomes that do not relate to the topics of health or stress (e.g., learning success or transfer of educational content related to the subject studied), outcomes that relate to teaching others
Formalities	Articles in English or German of all publication types, including empirical research (all study designs), reviews, and gray literature (e.g., discussion paper)	Abstracts, posters, whole anthologies, protocols, no full-text access

**Table 3:** Data extraction chart.

**Author**	Labrosse et al. [35]	Moffett et al. [36]	Sánchez-Ruiz et al. [37]	Yllana-Prieto et al. [38]
**Year**	2024	2023	2022	2021
**Country**	France	Ireland	Spain	Spain
**Study Design**	Cross-sectional (mixed methods)	Pretest-posttest (mixed methods)	Cross-sectional (mixed methods)	Pretest-posttest (quantitative)
**Primary Goal**	Describe the coproduction process of a deER through testing and adapting an existing ER	Describe the development process of a deER through testing a prototype ER	Analyze students’ opinions, feelings, and performances during and after playing a deER	Analyze how the deER affects multidimensional domains (e.g., attitudes, emotions) of students
**Target Group**	Healthcare students (first-year to PhD candidates of all health-related studies), University of Bordeaux, France	Second-year medical students, RCSI University of Medicine and Health Sciences, Ireland	First-year Aerospace Engineering students, course: Mathematics I, Technical University of Valencia, Spain	Senior-year students in Primary Education course: Knowledge of the natural environment, University of Extremadura, Spain
**Participants**	45	22	296	42
**Name of deER**	EscapeCovid	The hidden hospital	Ten short-duration deERs with no name	One deER with no name
**Type of deER**	Multiplayer (4–6 player), digital synchronous interaction	Multiplayer (4–5 player), digital synchronous interaction	Multiplayer (3–5 player), face-to-face synchronous interaction	Unclear, players connect to a virtual meeting, but play individually
**Technology**	deER developed by startup Tricky, online computer game using cameras and headphones for discussion; no details on programming software	Online game played via Microsoft Teams, using breakout rooms, web-conferencing, and screen sharing, developed with the interactive Genial.ly platform	Online game played on a digital platform in the same analog room, developed with Genial.ly platform and RPG Maker MZ software	Online game played individually via computer, tablet, or mobile; instructions on university’s Moodle platform; communication via email, Zoom videoconferencing
**deER Story**	Game set in Thomas’ room, a fictional university student in a shared flat, facing challenges of the first Corona lockdown	Students solve puzzles and escape a fictional creepy hospital, managing uncertainty in transitioning from classroom to clinical placement	No overarching fictional story, only individual games; students choose avatars with characteristics, and avatars are penalized/rewarded	Activity focuses on the universe, solar system, and the sun, content related to sustainable development; students receive instructions from a letter by Carl Sagan
**Quiz** **Elements**	Linear structure: Players can only move to another room if they solve all enigmas; three rooms, they solve puzzles by clicking on objects, which triggers a riddle	Ten puzzles (numerical, word-based, logic, general knowledge, three in-game reflections); participants can follow different pathways, one meta-puzzle to complete game	Linear structure: solving one task unlocks the next, including distracting, useless clues; requires specific knowledge or typical ER actions (e.g., finding hidden symbols)	Linear structure: solving one challenge leads to the next; includes, e.g., digital locks, digital puzzles, crossword puzzles
**Provided Support**	Group guided by a game guide, who explains rules and answers questions; final debriefing where participants share experiences	Support throughout the process: preliminary discussion, signposting strategy, technical support, debriefing	Students can request help if stuck, support offered to all groups if one group receives it, count of student attempts tracked in rooms, game provides clues and assistance	Trainer encourages interaction and involvement, active role in online sessions, and constant attention to participants’ doubts and concerns
**Learning Goals**	Promote students’ mental health literacy, beliefs about mental health, management of emotions, and positive coping strategies during the COVID-19 pandemic	Learn to manage uncertainty during transition from classroom to clinical placement, e.g., managing complex information, recognizing ambiguity, working with the different medical outcomes	Reinforce knowledge obtained by students and introduce new concepts regarding the syllabus of Mathematics I (calculus of several variables and series)	Teach content related to the universe (especially the solar system, the sun, and its role as a source of renewable energy for sustainable development)
**Health-Related Outcomes**	Understand the importance of discussing mental health, increase knowledge, and encourage open conversations about mental health, destigmatize it	Analyze the impact of gameplay on participants’ uncertainty tolerance (as a source of stress)	Analyze the predominant feelings during the escape room; evaluated feelings: positive, negative, neutral (e.g., stress)	Analyze students’ emotions before and after deER, positive and negative emotions (e.g., nervousness, frustration, concerns)
**Framework Used**	Co-creation process using the PRODUCES framework and a participatory methodology approach for the design phase	Design-based research approach and a guiding conceptual framework “Community of Inquiry”	No information on framework used for the design and development of the escape rooms is provided	No information on framework used for the design and development of the escape rooms is provided

**Table 4:** Corresponding knowledge requirements, learning, and gaming objectives.

**Identified Knowledge Requirements**	**Derived Learning Objective**	**Derived Gaming Objective** **and Implementation**
Level 1: Time management during studies “How can I manage my studies in a more organized and structured way?”	(1) Learn how to plan weekly and monthly goals. (2) Learn how to create a realistic schedule. (3) Learn how to prioritize tasks in a meaningful way. (4) Learn how to use breaks and free time effectively.	Starting in a virtual student apartment, the player should receive core time management strategies through tips hidden in collectible clocks. The core information is integrated in the form of tips in different hidden clocks as collectable items. The player should use the core information to prioritize classic tasks in the schedule. This prioritization is realized as a sorting task of different to-dos in a working document on the computer.
Level 2: Strategies and internal university offers for coping with stress “Which internal university offers and methods of mindfulness and meditation can I use in my everyday study life to cope with stress?”	(1) Learn which meditation and mindfulness strategies are available and whether these could be useful for everyday life. (2) Learn what internal university offers are available and what can be used. (3) Learn which resources are needed and available to cope with stress.	After arriving at the library to study, the player should learn about different methods of coping with stress. The methods and possibilities for coping with stress are recorded on index cards with various exercises to strengthen mindfulness and meditation in everyday study life, which are distributed in the library and can be read and collected. The player should also learn where they can find out about internal university topic-related offers. This information can be accessed once all the index cards have been collected and the laptop in the library has been decrypted.

**Table 5:** Characteristics of the sample of the pilot test (*n* = 4).

Variable	Result	Scale
Gender Ratio (female:male)	3:1	/
Age (mean in years)	26.25	/
Desired degree (name)	Master of Science in Digital Public Health: 4	/
Technical affinity (mean in points) ^1^	3.50	(1: “Does not agree at all” to 6: “Completely agree”)
Health consciousness (mean in points) ^2^	1.75	(−3: “I do not agree at all” to +3: “I completely agree”)
Stress level (mean in points)	3.78	(1: “Not stressed” to 7: “Greatly stressed”)
Experience with deER (yes:no)	1:3	/

^1^ Higher values indicate greater affinity for technology. ^2^ Higher values indicate greater consciousness about health.

**Table 6:** Results of the SUS—item mean and standard deviation (SD) (*n* = 4).

Question	Scale	Mean ± SD
Q1: I think that I would like to use this system frequently	Likert (1 to 5)	3.00 ± 2.68
Q2: I found the system unnecessarily complex	Likert (1 to 5)	1.50 ± 2.05
Q3: I thought the system was easy to use	Likert (1 to 5)	4.25 ± 3.82
Q4: I think that I would need the support of a technical person to be able to use this system	Likert (1 to 5)	1.75 ± 2.31
Q5: I found the various functions in this system were well-integrated	Likert (1 to 5)	4.00 ± 4.41
Q6: I thought there was too much inconsistency in this system	Likert (1 to 5)	2.25 ± 1.94
Q7: I would imagine that most people would learn to use this system very quickly	Likert (1 to 5)	3.75 ± 2.34
Q8: I found the system very cumbersome to use	Likert (1 to 5)	1.50 ± 2.01
Q9: I felt very confident using the system	Likert (1 to 5)	3.75 ± 2.96
Q10: I needed to learn a lot of things before I could get going with this system	Likert (1 to 5)	1.50 ± 2.07
T: Global SUS	SUS (0 to 100)	75.60 ± 11.6

## Data Availability

The original contributions presented in the study are included in the article, further inquiries can be directed to the authors. Data are contained within the article.
